# Evaluation of MENAQUINONE‐7 and fat‐soluble vitamin production by starter cultures during fermentation in dairy products using RPLC method

**DOI:** 10.1002/fsn3.4474

**Published:** 2024-10-26

**Authors:** Vildan Altuncu, Aykut Kaymaz, Bilge Ertekin Filiz, Ebru Çubuk Demiralay, Tuğba Kök Taş

**Affiliations:** ^1^ Department of Food Engineering, Engineering Faculty Suleyman Demirel University Isparta Turkey; ^2^ Department of Basic Pharmaceutical Sciences, Faculty of Pharmacy Suleyman Demirel University Isparta Turkey

**Keywords:** fat‐soluble vitamins, fermented dairy products, Menaquinone‐7, RPLC

## Abstract

Vitamin K2, also known as menaquinone, has become a significant research focus, particularly in fermented products. This study aims to investigate the content of menaquinone‐7 (MK‐7), an essential form of vitamin K, and other fat‐soluble vitamins (A, D, E) during the fermentation of various fermented milk products. The vitamin contents of six fermented milk products were analyzed: two yogurt samples (produced with commercial starter and probiotic starter), two kefir samples (produced with commercial starter and kefir grain), and milk fermented with *Lactobacillus acidophilus* or *Bifidobacterium bifidum*. Throughout the fermentation process, continuous pH monitoring was conducted, and fermentation was terminated based on pH levels. Fat‐soluble vitamins and vitamins K1, K2 were extracted from samples taken at specific fermentation periods and quantitatively determined using the reverse phase liquid chromatography (RPLC) method. The developed method was validated according to ICH guidelines. Simultaneously, the microbial content of the samples was analyzed. Among the fermented dairy products analyzed, the highest MK‐7 content (4.82 μg/100 g) was found in the kefir sample produced with kefir grain (KG). The diverse microorganisms in kefir grains necessitated detailed research to explain their role in this complex mechanism. In yogurt samples, the presence of *L. acidophilus* in the YB sample suggests that this bacterium may be responsible for the observed differences. This is supported by the LA sample, where production with *L. acidophilus* strain showed an increase from 0.97 to 1.70 at the 20th hour of fermentation. It was noted that the concentration of menaquinone‐7 increased throughout the fermentation period. Given the critical health effects of essential fat‐soluble vitamins and MKs, determining their content in commonly consumed fermented dairy products and understanding the influence of starter cultures, which are potential probiotics, on vitamin production underscores the importance of this research. It is important to highlight the potential of fermented products in nutritional recommendations.

## Graphical abstract

Vitamin K2 (menaquinone‐7, MK‐7) levels were measured during the fermentation of various dairy products. Six different fermented milk products were analyzed, including yogurt, kefir, and milk fermented with probiotics. Reverse phase liquid chromatography (RPLC) was used to quantify vitamin contents. The highest MK‐7 content (4.82 μg/100 g) was found in kefir made with natural kefir grain. MK‐7 levels increased during fermentation, while levels of other fat‐soluble vitamins (A, D, E) remained stable. The study emphasizes the health benefits of MK‐7 and the importance of probiotics in enhancing vitamin production in fermented dairy products.

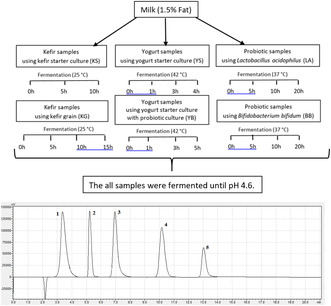

## 1 INTRODUCTION

Vitamin K is an indispensable cofactor, enhancing the synthesis of specific blood coagulation factors and proteins crucial for bone metabolism, vascular biology, and the prevention of osteoporosis (Frandsen & Gordeladze, 2017; Wen et al., 2018). Ongoing investigations explore the impact of vitamin K on energy metabolism, inflammation, and the risk of vascular calcification (Ahmed & Mahmoud, 2015; Beulens et al., 2013; Booth et al., 1993). Current U.S. recommendations suggest daily vitamin K intakes of 90 μg for women and 120 μg for men. Vitamin K, initially absorbed in the diet in quinone form, undergoes transfer to the blood. Phylloquinone (vitamin K1) and menaquinones (vitamin K2) are two biologically active forms. Menaquinones (MKs) with varying side chain lengths designated as MK‐n, indicate the number of unsaturated isoprenoid residues. Long‐chain menaquinones, like MK‐7 and MK‐10, are exclusively synthesized by bacteria. MK‐7, with an extended half‐life in human blood and high bioavailability, is also produced by various microorganisms (Gast et al., 2009; Geleijnse et al., 2004; Schurgers et al., 2007; Suttie, 1995). The exploration of bioprocess engineering technologies for industrial MK‐7 production has surged in the last decade (Berenjian et al., 2014). Since MK‐7 cannot be synthesized by human cells, its intake relies on fermented products, specific foods, or supplements. Despite vitamin K2, especially MK‐10, being produced by gut biota, its contribution to human vitamin K2 needs remains uncertain due to its unlikely absorption in the region of synthesis (Schurgers & Vermeer, 2000). Dietary sources of vitamin K2 are limited, earning it the moniker “platinum vitamin.” MK‐7 is derived from three main sources: plant extracts, animal foods or foodstuffs, and chemical synthesis or microbial production. Research on the vitamin K2 content of fermented foods is still limited (Ren et al., 2020; Szterk et al., 2018). Certain lactic acid bacteria, such as *Bacillus subtilis* natto, can produce MK‐7 during fermentation (Southee et al., 2016). Morishita et al. (1999) studied menaquinone production by lactic acid bacteria and found that strains like *Lactococcus lactis* subsp. *cremoris, Lactococcus lactis* subsp. *lactis*, and *Leuconostoc lactis* produced substantial amounts of MK‐7 to MK‐10 when grown in soymilk, as identified by high performance liquid chromatography.

There are various methods in the literature for the analysis of the fat‐soluble vitamins (A, D, E) and vitamin K selected in this study (Aturki & D'Orazio, 2005; Avan & Filik, 2021; D'Orazio et al., 2016; Suhara et al., 2005).Among these methods, the majority of analyses are carried out on different samples with the reverse phase liquid chromatography method (RPLC), which is a mode of the high‐performance liquid chromatography method (Alali et al., 2020; Aresta et al., 2020; Chen et al., 2022; Starek et al., 2023). The RPLC method is a well‐established method for the analysis of vitamin K in various matrices, including dairy products. Chromatographic separation of these vitamins was performed on a Kinetex C18 (250 × 4.6 mm I.D., 2.6 μm, 100 Å) column. This column was preferred because it provides a high degree of hydrophobic selectivity, reproducibility, and symmetrical peak shape compared to traditional C18 columns. The separation of five compounds in 14 minutes was achieved with good peak symmetry and reproducibility.

This study aims to investigate the production of MK‐7 in various dairy products during fermentation periods using the RPLC method. It also focuses on determining the amount of fat‐soluble vitamins during fermentation and revealing changes in MK‐7 concentration compared to other fat‐soluble vitamins. This article aims to provide valuable insights into the potential of dairy products as a source of MK‐7 during fermentation, thereby improving our understanding of the nutritional content of fermented dairy products and their impact on human health.

## 2 MATERIALS AND METHODS

In studies, MK‐7, a form of vitamin K2 produced by bacteria, is identified as a potential source in some fermented dairy products. While the main focus of this research is on MK‐7 in fermented products, it is considered whether the source is the starter cultures used. Therefore, two types of starter cultures were selected for different fermented products.

### 2.1 Bacterial cultures

The kefir grains used in the production of traditional kefir were provided by Danem Co., located in Technopark (Isparta, Turkey). The starter kefir culture for industrial kefir sample was obtained from CHR Hansen (Denmark) . Two types of yogurt cultures were produced: one using a yogurt starter culture containing *Lactobacillus bulgaricus* and *Streptococcus thermophilus* (0.1 g/100 mL; YoFlex, CHR Hansen) and the other using a probiotic yogurt culture (BüyüYo, Danem Co., Technopark) containing *Lactobacillus bulgaricus*, *Streptococcus thermophilus, and Lactobacillus acidophilus* (0.1 g/100 mL). For the development of fermented probiotic drink samples, freeze‐dried strains of *Lactobacillus acidophilus* (DSM No: 20,079) and *Bifidobacterium bifidum* (DSM No: 20,456) were procured from DSMZ (Deutsche Sammlung von Mikroorganismen und Zellkulturen, German Collection of Microorganisms and Cell Cultures).

### 2.2 Methods

#### 2.2.1 Production of fermented dairy products

To prepare active cultures, reconstituted milk (dry matter value: 12%) was pasteurized to 85°C for 15 min. Two types of kefir cultures were produced using kefir starter culture (0.1 gr/100 mL) and kefir grains (2 g/100 mL). Kefir cultures were fermented at 25°C up to pH of 4.6 was reached, and the completion times were recorded for both. . Two types of yogurt cultures were produced using a yogurt starter culture (0.1 g/100 mL) and probiotic yogurt starter culture (0.1 g/100 mL). Yogurt cultures were fermented at 42°C until pH 4.6 was reached, and both fermentation completion times were recorded. Stock cultures of *Lactobacillus acidophilus* (Code: ATCC 4356) and *Bifidobacterium bifidum* (Code: ATCC 29521) (Deutsche Sammlung von Mikroorganismen und Zellkulturen, German Collection of Microorganisms and Cell Cultures) stored at −20°C. After thawing, 100 μL of each culture was activated in MRS broth for 2 days at 37°C. The cultures were added at 1.5% to the reconstituted milk and incubated at 37°C until pH 4.6 was reached. The single probiotic cultures were prepared at 37°C up to pH 4.6.

**FIGURE 1:**
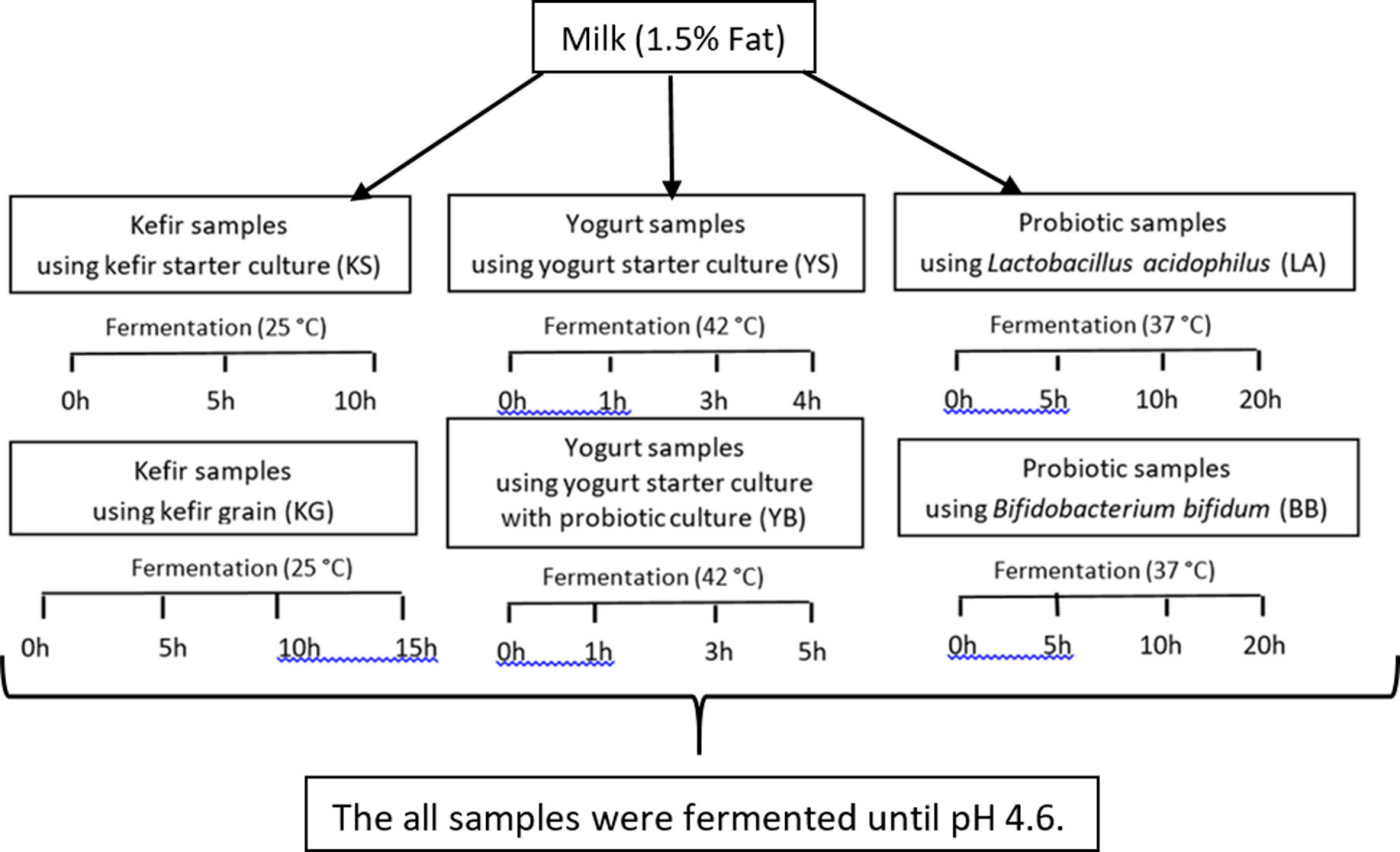
A chart showing the sample codes of three different fermented products in two different cultures and the timing of the samples taken during fermentation.

All samples from prepared cultures (2%) were analyzed using milk (1.5% fat, Sütaş Co). Only the fat content of the milk was standardized. The calculation for vitamin extraction was performed based on the fat content. Kefir samples were fermented at 25°C, probiotic samples at 37°C and yogurt samples at 42°C until pH 4.6 was reached Each fermented product was sampled at different times (Figure 1).

#### 2.2.2 Microbiological analyses

Certain bacteria do produce MK‐7 and other vitamins during their growth. For example, strains of lactic acid bacteria like Bacillus subtilis natto, Lactococcus lactis, and Leuconostoc species synthesize MK‐7 and other vitamins while fermenting food products. Microbiological analyses were conducted to determine the development and count of these bacteria. The *Lactobacilli* content was assessed by culturing in de Man, Rogosa, and Sharpe (MRS) medium (Merck) under anaerobic conditions (6% CO_2_) at 37°C for 3 days. Lactic streptococcal counts were determined using an M17 medium (Merck) at 37°C for 2 days. Yeasts were cultivated on potato dextrose agar (Merck), supplemented with 1.4% lactic acid, at 25°C for 5 days (Kök‐Taş et al., 2013).

#### 2.2.3 Extraction of fat‐soluble vitamin

This procedure was adapted from Salo‐Väänänen et al. (2000). A 30 g homogeneously sampled portion was mixed with 100 mL of ascorbic acid solution (15 g/L ethanol) and 25 mL of potassium hydroxide solution (125 g/100 mL). The saponification process was carried out through continuous shaking in a water bath at 60°C for 45 min. After cooling to room temperature, the samples were transferred to a separating funnel, and the upper phases were extracted three times with 50 mL of petroleum ether. To the combined extracts, 25 mL of distilled water was added, and the supernatant was separated once again. To eliminate any remaining water, 1 gram of sodium sulfate was introduced. The oil was extracted from the sample using a rotary evaporator (Heidolph Instruments, Laborota 4000) at 50°C. Subsequently, methanol (10 mL) was added to the remaining oil, which was filtered using a 0.45 μm syringe filter (Isolab) to prepare it for analysis.

#### 2.2.4 Preparation of standard solutions and calibration standards

Vitamin A (Sigma Chemicals) 50 μg/mL, vitamins E, K1 (Sigma Chemicals) and K2 (USP Reference Standard, 863–61‐6) at 10 μg/mL, and vitamin D (Sigma Chemicals) 15 μg/mL were prepared by dissolving in ethanol (96%, v/v, Merck). These stock solutions were stored in the dark at +4°C. Ethanol was used for the dilution of the standards. A calibration curve was constructed using an external calibration method to determine the linear concentration range of the vitamins.

#### 2.2.5 Linearity and sensitivity

Calibration was performed to accurately determine the concentration of the analyte in a sample. The external calibration method was applied to determine the linearity of the developed method. Peak area values were plotted against five different solution concentrations prepared by diluting the stock solutions of the vitamins to be quantitatively determined. The limit of detection (LOD) and limit of quantitation (LOQ) were calculated. These parameters were determined according to signal/to noise ratios of 3.3:1 and 10:1. The described method was linear for five compounds. The results meet the acceptance criteria according to ICH guidelines Q2 (R1), which state that the correlation coefficient value (*r*) must be >0.999 (ICH, 2005).

#### 2.2.6 Precision data of the method

To determine the precision of the developed RPLC method, intraday (repeatability) and interday (reproducibility) studies were conducted. Vitamin solutions were prepared at two different concentrations within the linear working range determined in the calibration. Independent solutions prepared at different concentrations were analyzed three times a day. These prepared solutions were kept in a refrigerator at +4°C, with air contact minimized. After the intraday analysis, the retention times and peak area values of the compounds were recorded in the analysis performed on the third day.

#### 2.2.7 Quantitative calculations

Quantitative calculations of vitamins in samples were made based on the external calibration method. Calibration curves were drawn using peak area values of compounds against different concentration values obtained by diluting the prepared stock vitamin standard solutions. The slope and intercept values of the linear function were used in the calculations.

#### 2.2.8 Accuracy data of the method

The accuracy and recoveries of the entire method, including the matrix effect, were evaluated with spiked samples. Samples containing dairy products that did not contain the analyzed vitamins were in supplemented with known amounts of vitamins. After the normal extraction procedure, the amount of added vitamins was measured and the recovery percentage was calculated. Three parallel samples were analyzed by spiking known vitamin concentrations (A: 0.50, 2.50 μg/mL; D: 0.05, 2.50.10^−3^ μg/mL; E: 0.50, 2.50 μg/mL, K1: 0.50, 2.50 μg/mL, K2: 0.10, 1.00 μg/mL) in dairy products.

#### 2.2.9 Chromatographic analysis

Shimadzu HPLC device (Kyoto, Japan) was used for chromatographic analysis of fat‐soluble vitamins. The system used consists of a diode array detector (SPD‐M20A), column oven (CTO‐10ASVP), pump (LC20AD), degasser (DGU‐20A3), and manual injection system. The rheodyne injection valve is equipped with a 20 μL sample loop. The compounds were monitored with a diode array detector at 330 nm for vitamin A, 230 nm for vitamin E, and 265 nm for vitamin D, K1, and K2, respectively. In this study, chromatographic separation was carried out in a methanol‐acetonitrile (95:5, v/v) binary mixture. The total analysis time for each run was 14 min. Separation was performed on a Kinetex C18 column (250 × 4.6 mm I.D., 2.6 μm, 100 Å, Phenomenex®) at 30°C with a flow rate of 0.8 mL/min. Since the fat‐soluble vitamins are lipophilic, this new generation column with a carbon loading of 12% was chosen for less retention in the column. The retention time (t_R_) for each vitamin was determined as the average of three separate injections. Capacity factors for each compound were calculated using the expression k = (t_R_ − t_0_)/t_0_. Dead time (t_0_) was measured by injecting uracil (Sigma Chemicals) solution (0.1% in mobile phase) into the studied mobile phase.

#### 2.2.10 Statistical analysis

All statistical tests were performed using Microsoft Excel with the data analysis tool package. Origin version 8.0 statistical software (Origin Lab Inc.) was used to analyze the HPLC data.

## 3 RESULTS AND DISCUSSION

Vitamin K encompasses a group of fat‐soluble vitamins, including vitamin K1 (phylloquinone; PK), vitamin K2 (menaquinone; MK), and vitamin K3 (menadione). These vitamins are essential for bodily functions and, except for vitamin D, cannot be synthesized by the body. Bacteria use menaquinones (MKs) to shuttle electrons in the electron transport chain. They produce MKs via two metabolic pathways: a traditional pathway that converts chorismate to MK using a series of enzymes (MenA–MenG), prevalent in many gram‐positive microbes, and an alternative futalosine pathway found in some gram‐negative bacteria and Archaea. In food production, gram‐positive bacteria are commonly used for fermentation (Hiratsuka et al., 2008). In this study, while determining the fat‐soluble vitamins in some fermented products, we also obtained results regarding the microbiological content of the samples.

The microbiological contents of samples during fermentation periods are given in Table 1. Lactobacilli counts were detected in the kefir and yogurt samples produced using different starter cultures. As fermentation time increased, there was a rise in the number of LAB and yeasts. Nche et al. (1994) reported that the increase in acidity and decrease in pH in kenkey were linked to the rise in LAB counts. In this study, as LAB counts increased, acidity increased, and the process was terminated at a product pH of 4.6. It was determined that the probiotic‐added yogurt had the highest LAB count, with a value of 10.51 log CFU/g. No yeast growth was observed in the samples except for the kefir samples. The number of cocci, which was initially about 1 log, was not observed in LA and BB samples in the later stages of fermentation.

**TABLE 1:** Microbial content of fermented dairy products (log cfu/ml).

Dairy products	Codes	Fermentation times (hour)	*Lactobacillus* spp.	Cocci	Yeast
Kefir	KS	0	1.86 ± 0.05	1.02 ± 0.05	–
		5	5.45 ± 0.02	3.43 ± 0.06	1.33 ± 0.11
		10	7.98 ± 0.19	6.89 ± 0.04	2.09 ± 0.09
	KG	0	1.86 ± 0.05	1.02 ± 0.05	–
		5	3.62 ± 0.03	4.05 ± 0.04	1.56 ± 0.08
		10	8.75 ± 0.14	8.95 ± 0.07	3.29 ± 0.07
		15	9.89 ± 0.08	9.77 ± 0.06	4.75 ± 0.07
Yogurt	YS	0	1.86 ± 0.05	1.02 ± 0.05	–
		1	2.12 ± 0.02	3.24 ± 0.03	–
		3	3.84 ± 0.01	4.06 ± 0.05	–
		4	7.54 ± 0.06	6.29 ± 0.11	–
	YB	0	1.86 ± 0.05	1.02 ± 0.05	–
		1	2.95 ± 0.11	3.02 ± 0.13	–
		3	8.45 ± 0.07	8.65 ± 0.07	–
		5	10.51 ± 0.05	9.62 ± 0.05	–
Fermented with *Lacidophilus*	LA	0	1.86 ± 0.05	1.02 ± 0.05	–
		5	3.85 ± 0.04	–	–
		10	6.45 ± 0.12	–	–
		20	9.53 ± 0.02	–	–
Fermented with *B.bifidum*	BB	0	1.86 ± 0.05	1.02 ± 0.05	–
		5	2.87 ± 0.05	–	–
		10	5.68 ± 0.02	–	–
		20	8.89 ± 0.70	–	–

*Note*: Values (mean ± standard deviation).

According to research by Kök‐Taş et al. (2013), the *Lactobacilli* counts in kefir samples fermented with kefir grains and starter culture were approximately 9.21 and 9.27, respectively, after 24 h of fermentation. In the same study, *Lactococcus* counts were around 9.23 and 9.29, while yeast counts measured approximately 5.50 and 4.77 for grain and starter‐fermented kefir samples. In another study by Rosa et al. (2017), after a 15 h fermentation period, there were approximately 7–8 log units of lactobacilli and lactococci, with no significant difference observed between the 15 and 22 h marks at the end of fermentation. Similarly, in Brazilian kefir, as reported by Leite et al. (2013), the numbers of *Lactobacilli* and *Lactococci* remained at around 10 log cfu/g between the 10–18 h of fermentation.

Bergamo et al. (2003) suggested that variations in microbiological content during fermentation may impact the levels of vitamins A, D, E, and K in dairy products. In this study, while the synthesis of vitamins A and D is relatively low during fermentation as observed in   , the content of vitamins E and K, particularly vitamin K2, can be influenced by the bacterial community composition during this process. Throughout fermentation, the counts of Lactic Acid Bacteria (LAB), cocci, and yeast, as well as the concentrations of vitamins A, D, E, and K , increased in all samples.

The microbiological composition of dairy products during fermentation plays a crucial role in the formation of vitamin K2, with specific bacterial strains being key contributors to its production (Kang et al., 2022). The values of vitamins A, D, and E in the products were determined to originate from milk, and these vitamin levels continued to increase during the fermentation period. Certain bacteria present in kefir have been found to synthesize vitamin K, encompassing both K1 and K2 (Tarvainen et al., 2019). According to Walther et al. (2013), specific LAB species, notably *Lactococcus lactis*, commonly used in the industrial fermentation of various dairy products, can produce menaquinone through biosynthesis. Vitamin K2, a fat‐soluble vitamin crucial for bone and cardiovascular health, is produced by certain bacteria, including *B. bifidum*, and is found in specific fermented foods like natto (Yanagisawa & Sumi, 2005).

It is important to note that the impact of microorganisms on fat‐soluble vitamin content in fermented foods can vary based on fermentation conditions and the composition of the milk used. Enzymes such as lipases and esterases, produced by microorganisms, play a role in releasing fat‐soluble vitamins A, D, E, and K from milk fat globules, thereby increasing their accessibility to the human body.

In this study, qualitative and quantitative determinations of fat‐soluble vitamins A, D, E, and K were carried out using the RPLC method. Since the compounds are hydrophobic, a mixture of methanol and acetonitrile (95:5, v/v) was used as the mobile phase for chromatographic separation. Acetonitrile and methanol, which have high elution power, are frequently preferred solvents in RPLC studies, although they may pose some problems in terms of health safety and ecological impact. In this study, methanol was used at 95% (v/v) in the mobile phase because it is biodegradable and less toxic than acetonitrile. Chromatographic separation of the selected vitamins was carried out on a Kinetex C18 (250 × 4.6 mm I.D., 2.6 μm, 100 Å, Phenomenex®) column. This column is a core‐shell product that can provide better separation power than classical C18 columns for ionizable and neutral compounds with hydrophobic and hydrophilic properties. In this study, the t_R_ value of each selected vitamin was determined in triplicate. Accordingly, the percentage of relative standard deviation (%RSD) of the retention times of the compounds is below 2%, indicating that the change in reproducible injections is minimal. For the quantitative determination of these compounds, the capacity factor (k) values must be in the range of 1 ≤k ≤10, the selectivity factor (α) must be ≥1.15 and the separation factor (R_s_) value must be ≥1.5. The α value is calculated by dividing the k_2_ value of the second peak by the k_1_ value of the first peak. The R_s_ value was calculated using the Purnell equation and the obtained data are given in Table 2.

**TABLE 2:** Calculated chromatographic data of compounds at optimum separation condition.

Compounds	t_R_	*N*	*k*	*α*	k_2_/1 + k_2_	α‐1/α	1/4√N	R_s_
Vitamin D	3.571	8200	1.215					
Vitamin A	5.311	9155	2.295	1.888	0.696	0.470	95.682	7.837
Vitamin E	7.022	10,655	3.356	1.463	0.770	0.316	103.223	6.288
Vitamin K1	10.372	8894	5.434	1.619	0.845	0.382	94.308	7.615
Vitamin K2	13.103	11,714	7.128	1.312	0.877	0.238	108.231	5.640
t_o_ (uracil)	1.612							

**FIGURE 2:**
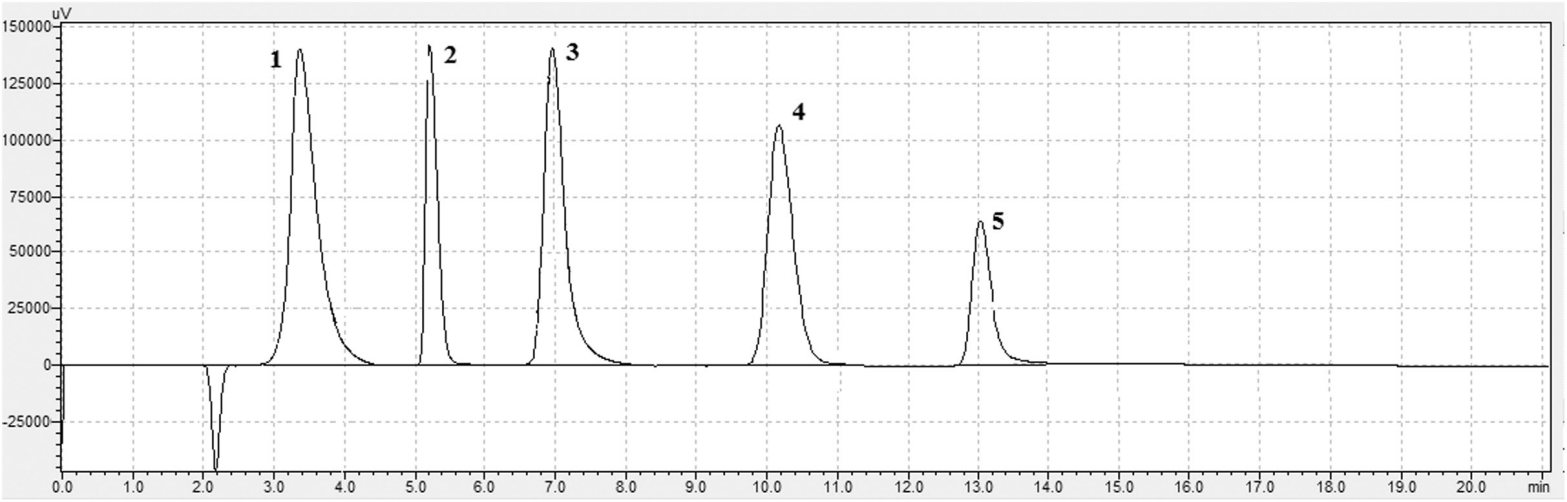
Standard mixture chromatogram (1)vitamin D (2)vitamin A (3)vitamin E (4)vitamin K1 (5)vitamin K2 (MK‐7).

As seen in Table 2, the theoretical plate number (N) which shows the efficiency of the selected RPLC column is over 2000. The calculated R_s_ values show that the compounds are separated from each other. Additionally, it was determined that the peak symmetry of the compounds was much better than traditional columns. The chromatogram obtained under the determined optimum separation conditions is shown in Figure 2.

Following the optimization of the liquid chromatographic method developed in the study, the method was validated for the quantitative determination of compounds. The obtained linear regression parameters are given in Table 3. According to Table 3 , the correlation coefficient (r) >0.999 is closely related to the accuracy of the analysis performed. It shows that the method is linear in the given concentration range for each vitamin. The lowest detection limit (LOD) and the highest limit of quantification (LOQ) for which reproducible results were obtained were for vitamin D and vitamin K1. The developed HPLC method can be considered sufficiently sensitive.

**TABLE 3:** Calibration data of studied compounds.

Calibration data	Vitamin A	Vitamin D	Vitamin E	Vitamin K1	Vitamin K2 (MK‐7)
Linearity range (μg/mL)	0.1–5.0	2.5.10^−5^‐0.1	0.25–5.0	0.25–5.0	0.05–2.0
Slope (μg/mL)	13.465	440.240	116.393	24.555	211.585
Intercept (μg/mL)	1130.1	1710.7	1408.6	−4726.2	−8213.3
r	1.000	1.000	0.999	0.999	1.000
LOD (μg/mL)	0.011	4.6.10^−6^	0.049	0.065	0.012
LOQ (μg/mL)	0.038	1.5.10^−5^	0.162	0.217	0.041

Repeatability is a measure of the precision of the method and can be calculated experimentally. The precision of the method was determined by taking intraday and interday repeatability measurements. For this, repeatability was calculated and evaluated for three independent standard solutions of vitamins at two concentration levels (μg/mL) from the linear calibration range, on the same day and 3 days later. The mean vitamin concentration did not differ significantly and was close to the expected value. The % RSD was calculated to evaluate the precision of the method developed for this study. The measured concentration and RSD% values calculated using the calibration function based on these data are provided in Table 4. The % RSD values of the results calculated from the analyses are below 2%, indicating good precision of this method..

**TABLE 4:** Intraday and interday precision results of the analysis method of compounds.

Compounds	Theoretical concentration (μg/mL)	Intraday measured concentration mean (μg/mL)	%RSD	Interday measured concentration mean (μg/mL)	%RSD
Vitamin A	0.5	0.501	0.798	0.511	1.174
	4.0	4.055	1.036	4.102	1.243
Vitamin D	2.50.10^−4^	2.42.10^−4^	0.679	2.55.10^−4^	1.176
	0.05	0.053	0.977	0.048	1.042
Vitamin E	0.5	0.503	0.815	0.521	1.171
	4.0	3.905	0.896	4.006	1.747
Vitamin K1	0.5	0.493	1.014	0.496	1.129
	4.0	4.038	0.495	3.906	0.768
Vitamin K2 (MK‐7)	0.1	0.103	0.485	0.096	0.625
	1.5	1.506	0.352	1.495	0.602

Accuracy was calculated by adding standard solutions at specific concentrations to dairy products. Samples were spiked at two different concentrations for each vitamin. The analysis was performed three times for each concentration. The following formula was used to calculate the average recovery: % recovery = (amount found/amount added) × 100. The calculated percentage recovery values are given in Table 5. The recovery of vitamins from the samples varied between 94.23% and 110.80% (average values), while the RSD varied between 0.84% and 2.34%. Therefore, it was concluded that the extraction method and analytical procedure performed in this study completely dissolved the vitamins in the matrices analyzed and the recovery of the compounds was high.

**TABLE 5:** Recovery values (%) for studied vitamins in dairy products.

Compounds	Spiking level (μg/mL)	Kefir		Yogurt		*Fermented with Lacidophilus*		*Fermented with B.Bifidum*	
		Mean recovery (%)	RSD (%)	Mean recovery (%)	RSD (%)	Mean recovery (%)	RSD (%)	Mean recovery (%)	RSD (%)
Vitamin A	0.50	98.60	1.81	100.60	1.64	103.45	0.94	108.20	1.88
	2.50	99.04	1.03	100.64	1.92	101.33	1.86	101.32	1.94
Vitamin D	2.50.10^−3^	98.40	1.06	100.40	0.98	101.20	1.21	102.40	2.11
	0.05	96.00	2.04	102.00	1.03	106.00	1.55	96.00	1.44
Vitamin E	0.50	103.20	1.87	104.40	1.41	95.16	1.48	97.80	1.16
	2.50	99.88	1.94	101.32	2.11	99.60	1.66	96.12	1.21
Vitamin K1	0.50	104.40	1.35	100.60	1.35	98.20	2.09	102.80	1.66
	2.50	99.88	1.77	110.80	1.84	101.76	2.15	101.24	2.01
Vitamin K2 (MK‐7)	0.10	97.00	1.26	97.94	1.02	106.19	1.78	96.50	2.34
	1.00	102.30	2.01	107.72	0.84	94.23	0.85	99.10	0.93

The developed chromatographic method was applied for the analysis and quantitative determination of the samples. The results obtained at different fermentation times are given in Table 6. Some samples did not provide results for vitamins below the linear operating range.

**TABLE 6:** Quantitative results of studied samples.

Dairy products	Codes	Fermentation times (hour)	Vitamin A (μg/100 g)	Vitamin D (μg/100 g)	E vitamin (μg/100 g)	K1 vitamin (μg/100 g)	MK‐7 (μg/100 g)
Kefir	KS	0	15.79 ± 0.02^a^	0.55 ± 0.02	1.11 ± 0.03	4.81 ± 0.07	0.97 ± 0.01
		5	23.62 ± 0.07	2.20 ± 0.03	1.69 ± 0.03	5.98 ± 0.04	1.14 ± 0.02
		10	26.65 ± 0.10	4.14 ± 0.02	2.08 ± 0.05	5.81 ± 0.05	1.47 ± 0.02
	KG	0	15.79 ± 0.06	0.55 ± 0.03	1.11 ± 0.02	4.81 ± 0.04	0.97 ± 0.02
		5	21.53 ± 0.02	5.24 ± 0.02	2.38 ± 0.05	5.85 ± 0.02	1.04 ± 0.06
		10	26.88 ± 0.02	2.70 ± 0.02	2.68 ± 0.03	8.24 ± 0.01	2.91 ± 0.03
		15	30.54 ± 0.05	4.27 ± 0.03	2.76 ± 0.03	8.84 ± 0.09	4.82 ± 0.05
Yogurt	YS	0	15.79 ± 0.11	0.55 ± 0.01	1.11 ± 0.02	4.81 ± 0.03	0.97 ± 0.02
		1	19.80 ± 0.08	1.41 ± 0.03	1.84 ± 0.02	4.81 ± 0.02	1.66 ± 0.05
		3	23.99 ± 0.03	3.06 ± 0.02	2.58 ± 0.06	8.24 ± 0.06	1.94 ± 0.04
		4	26.46 ± 0.04	3.21 ± 0.04	2.58 ± 0.02	7.22 ± 0.05	3.02 ± 0.05
	YB	0	15.79 ± 0.07	0.55 ± 0.03	1.11 ± 0.05	4.81 ± 0.06	0.97 ± 0.01
		1	24.21 ± 0.02	1.40 ± 0.02	1.83 ± 0.02	4.81 ± 0.05	1.79 ± 0.04
		3	26.98 ± 0.04	1.64 ± 0.03	2.20 ± 0.01	6.53 ± 0.02	3.01 ± 0.06
		5	26.36 ± 0.10	1.30 ± 0.01	2.20 ± 0.03	12.21 ± 0.06	3.82 ± 0.08
Fermented with *L. acidophilus*	LA	0	15.79 ± 0.05	0.55 ± 0.02	1.11 ± 0.02	4.81 ± 0.04	0.97 ± 0.02
		5	15.80 ± 0.04	2.33 ± 0.05	0.71 ± 0.02	9.94 ± 0.01	1.29 ± 0.04
		10	15.87 ± 0.02	2.27 ± 0.01	1.09 ± 0.03	9.92 ± 0.06	1.27 ± 0.03
		20	15.97 ± 0.01	4.31 ± 0.01	1.39 ± 0.04	9.93 ± 0.03	1.70 ± 0.02
Fermented with *B. bifidum* BB	BB	0	15.79 ± 0.08	0.55 ± 0.06	1.11 ± 0.05	4.81 ± 0.05	0.97 ± 0.01
		5	15.53 ± 0.08	2.49 ± 0.04	1.64 ± 0.03	8.25 ± 0.02	1.04 ± 0.02
		10	15.86 ± 0.06	2.93 ± 0.02	1.70 ± 0.02	11.60 ± 0.05	1.13 ± 0.04
		20	15.92 ± 0.04	2.54 ± 0.02	1.79 ± 0.06	12.58 ± 0.04	1.29 ± 0.02

Abbreviations: BB, Fermented probiotic drink with *Bifidobacterium bifidum*; KG, Kefir produced with kefir grains; KS, Kefir produced with the starter culture; LA, Fermented probiotic drink with *Lactobacillus acidophilus*; YB, Yogurt produced with probiotic culture; YS, Yogurt produced with the starter culture.

^a^ Confidence interval of the arithmetic mean with a probability of 95%.

When evaluating the relationship between Table 1 and Table 6, it was determined that fat‐soluble vitamins are present in certain amounts in milk. Additionally, in fermented samples, these vitamins increased during fermentation, and this increase correlated with the bacterial count as shown in Table 1. According to Table 6, kefir produced with kefir grains reached its highest value of 4.82 μg/100 g at the 15th hour. The diverse microorganisms in kefir grains necessitated detailed research to explain their role in this complex mechanism. In yogurt samples, the presence of *L. acidophilus* in the YB sample suggests that this bacterium may be responsible for the observed differences. This is supported by the LA sample, where production with the *L. acidophilus* strain showed an increase from 0.97 μg/100 g to 1.70 μg/100 g at the 20th hour of fermentation. Erdoğan and Ertekin Filiz (2023) analyzed menaquinone 4 (MK‐4) and menaquinone 7 (MK‐7) from K2 vitamins in fermented cabbage samples in their study. They determined the content of MK‐4 to be 1.00–4.63 μg/100 g and MK‐7 to be 5.55–14.48 μg/100 g in pickled cabbage samples. Fu et al. (20177) determined the amount of vitamin K2 in dairy products to be 5.1–38.1 μg/100 g. Manoury et al. (2013) examined the contents of MK‐7, MK‐8, MK‐9, and MK‐10 in various dairy products.

In a Finnish study quantifying carotenoid concentrations in various dairy products, β‐carotene contents ranged from 3.0 to 186.5 μg/100 g (Ollilainen et al., 1989). LC–MS and LC–MS/MS analysis of whole milk and fresh cow's milk revealed vitamin D‐3 concentrations of 0.2 μg/L and 0.5–0.6 μg/L, respectively (Schmid & Walther, 2013). α‐Tocopherol contents in dairy products with different fat contents were found to be in the range of 4.5–45.5 μg/100 g (Kaushik et al., 2001). Elder et al. (2006) reported that vitamin K contents in various animal products, including dairy products contained K1 and MK‐4 in the range of 0.2–3.0 and 0.4–10.4 mg/100 g, respectively.

Two different cultures were used in the production of kefirs, and the choice of kefir cultures had an impact on vitamin values. Kefir samples produced with a kefir starter culture reached a pH of 4.6 in 15 hours, while kefir made with kefir grains achieved this pH level in 24 hours. All vitamins, including Vitamin K1 and MK‐7, increased during fermentation. The kefir starter culture contains *Debaryomyces hansenii, Leuconostoc, Streptococcus thermophilus, Lactobacillus lactis, Lactobacillus diacetylactis*, and *Lactobacillus cremoris*, while kefir grains a rich diversity of lactic acid bacteria, acetic acid bacteria, and yeasts (Erdogan et al., 2019).

Fat‐soluble vitamins increased during fermentation in both kefir samples produced with kefir starter culture (KS) and kefir grains (KG). There is no existing publication specifically addressing the effect of microorganisms on the increase of vitamins A, D, and E. A,t the 10th hour, the Vitamin K1 and MK‐7 contents of the KS sample were determined to be 5.81 and 1.47 μg/100 g, respectively, with a significant increase observed. Similarly, for the KG sample, which exhibited a much richer microorganism diversity, the values were 8.84 and 4.82 μg/100 g, respectively, and they increased significantly by the 15th hour of fermentation.

The situation was similar in yogurt samples prepared using two distinct cultures. The yogurt sample produced using yogurt starter culture (*Lactobacillus bulgaricus* and *Streptococcus thermophilus*) achieved pH 4.6 in 4 h, while the yogurt sample made with probiotic yogurt starter culture (*Lactobacillus bulgaricus* *Streptococcus thermophilus*, and *Lactobacillus acidophilus*) reached pH 4.6 in 5 h. An increase in all vitamins, including fat‐soluble vitamins, was observed during fermentation in both YS and YB samples. Similar observations were noted for Vitamin K1 and MK‐7. In examining the vitamin K1 and MK‐7 values, the YS sample measured at the 4th hour yielded 7.22 and 3.02 μg/100 g, respectively. Meanwhile, the YB sample, with probiotic cultures, exhibited a microorganism diversity of 12.21 and 3.82 μg/100 g at the 5th hour, indicating a difference attributed to the addition of *Lactobacillus acidophilus* and distinct strains of other yogurt cultures..

Additionally, the vitamin production of probiotic cultures alone was scrutinized. *Lactobacillus acidophilus* reached pH 4.6 after 20 h, and vitamin values, including Vitamin K1 and MK‐7, increased during fermentation. Vitamin K1 was detected at 4.81 μg/100 g in the early hours for all the samples it was determined to be 9.94 μg/100 g at the 5th hour and exhibited a continuous increase over 20 h. The MK‐7 value was measured at 1.27 μg/100 g at the 10th hour and further increased to 1.70 μg/100 g at the 20th hour.

*Bifidobacterium bifidum*'s vitamin production was assessed as a standalone probiotic culture. Bifidobacterium bifidum attained pH 4.6 after 20 hours, and vitamin values, including Vitamin K1 and MK‐7, increased throughout fermentation. At the 5th hour, Vitamin K1 was found to be 8.25 μg/100 g, with MK‐7 at 1.04 μg/100 g; however, MK‐7 did not change at other time points.

Studies on menaquinone production in various fermented products, such as the research conducted by Sungur et al. (2021), revealed significant MK‐4 and MK‐7 contents in kefir, measuring 90.68 ± 0.16 and 26.96 ± 0.10, respectively. Certain lactic acid bacteria strains, considered Generally Recognized as Safe (GRAS), play a crucial role in menaquinone production. For instance, Propionibacteria, such as those found in Norwegian Jarlsberg cheese and Swiss Emmental cheese, produce MK‐9 as a major menaquinone (Qureshi et al., 2013). The absorption of menaquinone from dairy products, including MK‐4, MK‐7, MK‐8, and MK‐9, is reported to be considerably better than phylloquinone absorption from vegetables. Fermented soybean foods like natto are also rich in menaquinones, particularly MK‐7.

Hartling (2014) reported that fermenting *with S. thermophilus, L. bulgaricus*, and *L. cremoris* leads to a yogurt‐like product that contains significantly high levels of menaquinones, with concentrations reaching up to 234 ng/g MK‐9 and 155 ng/g MK‐7. These values represent a substantial increase compared to those typically seen in yogurt. For instance, Kamao et al. (2007) found only 1 ng/g MK‐7 in yogurt, and Schurgers and Vermeer (2000) reported 2 ng/g MK‐9. The menaquinone concentrations observed in our study are more akin to those found in cheeses, as reported in studies by Schurgers and Vermeer (2000) and Manoury et al. (2013).

Manoury et al. (2013) conducted one of the most extensive surveys of menaquinones in cheeses, finding MK‐7 levels ranging from 0 to 223.3 ng/g and MK‐9 levels from 0 to 939.7 ng/g. Although these values are higher than the highest concentrations we detected, the levels of menaquinones in our samples that contained *L. cremoris* were greater than many of the cheeses reported in their study. In fact, the total menaquinone content in our ST + LB + LC sample with a 24 h delay in *S. thermophilus* and *L. bulgaricus* inoculation could be a significant source of dietary menaquinones. For example, a 100‐g serving of this yogurt‐like product could provide more than 49% of the Recommended Daily Intake (RDI) for vitamin K in Canada, which is 80 μg (including an unspecified amount of MK‐8; Canadian Food Inspection Agency, 2016).

Vermeer et al. (2018) emphasized the impact of microflora, ripening, and fat content on the MK content of Dutch cheeses. Fresh cheeses were reported to have lower menaquinone content compared to ripened ones, with a notable increase in long‐chain menaquinone levels observed in more mature cheeses after 13 weeks of ripening, attributed to bacterial growth during fermentation.

## 4 CONCLUSION

In this study, the amounts of fat‐soluble vitamins, especially menaquinone‐7, produced in different cultures during fermentation in some dairy products were determined by the RPLC method. The impact of bacteria on MK‐7 production was investigated throughout the fermentation process. The increase in fat‐soluble vitamins during fermentation in different fermented products may be due to varying culture contents. However, to confirm this, it is necessary to investigate each strain individually. In the study, it was found that kefir produced with kefir grains reached its highest value, indicating that approximately 12 mg/250 mL of MK‐7 can be obtained from a daily glass of kefir. However, the diverse microorganisms in kefir grains necessitate detailed research to explain their role in this complex mechanism.

Fermented products are widely acknowledged as significant sources of menaquinones (MK) due to bacterial action. The use of lactic acid bacteria as starter cultures in many fermented products, which are generally recognized as safe, is significant. This research contributes to similar studies investigating the production of MK by these bacteria, whose health benefits are increasingly recognized. Recent research has underscored the association between vitamin K2 deficiency and cardiovascular diseases, highlighting the importance of investigating the menaquinones produced by microbial cultures used in fermented products and those naturally occurring in dietary fermented items. Given that the daily requirement for vitamin K is approximately 1 μg per kilogram of body weight, it is important to highlight the potential of fermented products in nutritional recommendations.

## CONFLICT OF INTEREST STATEMENT

The authors declare that they have no known competing financial interests or personal relationships that could have influenced the work reported in this paper.

## Data Availability

The data that support the findings of this study are available on request from corresponding author. The data are not publicly available due to privacy or ethical restrictions.
